# Sociopragmatic pronouns in Limburgian: inferring speakers’ agency from self-reported automaticity, attitudes, and metalinguistic awareness

**DOI:** 10.1515/cog-2023-0141

**Published:** 2025-01-10

**Authors:** Joske Piepers, Ad Backus, Jos Swanenberg

**Affiliations:** Department of Culture Studies, Tilburg School of Humanities and Digital Sciences, Tilburg University, Warandelaan 2, 5037 AB Tilburg, The Netherlands; Meertens Institute, Royal Netherlands Academy of Arts and Sciences (KNAW), Oudezijds Achterburgwal 185, 1012 DK Amsterdam, The Netherlands

**Keywords:** agency, language contact, non-feminine pronoun, self-reported language use, variation

## Abstract

How much of everyday language use takes place on autopilot, how much are speakers aware of, and how do their attitudes relate to this? In particular, how do these factors together account for variation between speakers? Limburgian, a regional language within the Netherlands, is under pressure from Dutch in an intensive language contact situation. The use of a non-feminine subject pronoun for women is a Limburgian feature which is not shared with Dutch. Limburgian speakers show a large range of variation regarding this feature, both when it comes to its use, and how it is perceived. By studying speakers’ self-reports of three concepts – automaticity, attitudes, and metalinguistic awareness – as well as how these together relate to self-reported language use (*N* = 405), this paper investigates to what extent speakers have control over their own language use. Our findings suggest that self-reported automaticity is the driving force in the use of the non-feminine pronoun, but also that this autopilot may be curbed by metalinguistic awareness and attitudes. Importantly, speakers vary considerably on all three concepts, highlighting once more that language users are not a monolith, and that individual speakers may react differently in a language contact situation.

## 1 Introduction

It is no coincidence that there are sub-disciplines of linguistics dedicated to cognition and to sociality: as our main tool for communication, language needs both cognitive skill and social usefulness. Its use is thus a constant interplay between automaticity and agency. That is, on the one hand, language production is strongly dependent on automatic processes because of how swiftly it occurs, but at the same time, speakers can deliberately choose how to say something in a way that best fits their communicative intentions (Campbell-Kibler 2016; Christiansen and Chater 2016; Nycz 2016). The trade-off between agency and automaticity, however, is difficult to ascertain, as separating them is challenging when examining speakers’ linguistic output. For example, in the literature on bilingual codeswitching, it has been shown that bilinguals may switch on purpose, to display their multilingual identity (agency), or inadvertently, because a word in the other language was more readily activated for them (automaticity). The result is in both cases a code-switch, but with very different underlying causes (cf. Barking et al. 2024; Sharma 2018).

Automaticity in language use may be defined as the production of language without consciously attending to it much. It is clear from decades of psycholinguistic and sociolinguistic research that while speaking, we vary between high and low degrees of conscious attention to speech, that automaticity is therefore a gradient phenomenon, and that many linguistic and social factors play a role in determining the degree of automaticity at any point in a speaker’s utterance (Christiansen and Chater 2016; Kristiansen 2010; Labov 1972). Some aspects of language are, in all probability, automatically activated most of the time (e.g., inflectional morphology), whereas others are much more likely to be consciously selected (e.g., ‘the right word for the concept’, or items which serve to mark that a speaker identifies with a certain group; Johnstone 2016). Most likely, a high degree of automaticity is typical when a speaker does not have to make a choice between two or more variants, while variation is a natural trigger for the act of choosing or selecting. The reality, however, is considerably less black-and-white: any linguistic element can be produced either with or without much conscious attention, and automaticity is itself a gradual concept rather than ‘all-or-none’ (Hartsuiker and Moors 2017: 207; Moors 2016).

More importantly, meanings may be verbalized in various ways (favoring conscious selection) and such a choice may serve social identity marking functions (further favoring conscious selection), but still occur frequently enough to have also produced considerable degrees of cognitive entrenchment or habitualization (favoring automatic activation; Bybee 2010). Individual speakers may differ from one another (i.e., they make different choices, have different entrenchment levels, and entertain different social sensitivities regarding the verbalizations in question; Dąbrowska 2008, 2012]), and their motives may vary between different communicative contexts (e.g., speakers may be more or less on automatic pilot, or be more or less worried about ‘sounding right’; Bijvoet and Fraurud 2015; Harder 2014). A usage-based approach to language advocates for recognizing these sources of variation (or stability) as inherent characteristics of language use, as well as the general contribution of both automatic production and deliberate choice. As such, it urges a closer integration of the social and cognitive mechanisms that drive speakers’ language use (Backus 2021; Dąbrowska 2020; Hakimov and Backus 2021). With this paper, we aim to contribute to that goal.

### 1.1 Sociopragmatic pronoun use in Limburgian

Our study focuses empirically on the use of a specific pronoun in Limburgian. Traditionally, like in related West-Germanic dialect varieties (for discussions see Busley and Nübling 2021; Martin 2019; Nübling 2015), Limburgian has two options for referring to women: the feminine pronoun *ziej* ‘she’, and the neuter pronoun *het* ‘she’ (lit. ‘it’). The use of these pronouns is generally sociopragmatically determined, i.e., the choice between the feminine or the neuter pronoun seems largely based on an assessment of the interpersonal relationship between the speaker and the woman they refer to (see Piepers et al. 2021, 2023b for more details, and Aikhenvald 2016; Steriopolo 2021 for such phenomena in general). The sociopragmatics of Limburgian third-person pronouns for female referents are somewhat similar to those of second person pronouns in various European languages (French *tu*/*vous*, Spanish *tú*/*usted*, German *du*/*Sie*, etc.), where a T/V-distinction is made based on solidarity, relative age and emotional closeness. Furthermore, T-pronouns are used for intimate communication in informal contexts, whereas V-pronouns are used to express politeness and respect in more formal contexts (Brown and Levinson 1987; Kretzenbacher et al. 2006; cf. Bakker 1992: 10: “there is a clear parallelism and direct connection with the second person singular”). Previous research suggests an ‘age-constraint’ restricting the use and appropriateness of Limburgian *het*: overall, speakers used *het* significantly more often for younger women than for older ones, and they judged *het* as significantly more acceptable for younger women than for older women. Many speakers appear aware of this pattern, and can reflect on it, as well as on the sociopragmatic aspects of the pronoun and its use (Piepers et al. 2023b). This confirms observations made by earlier researchers that the pronoun *het* is generally associated with personal closeness and youthfulness (e.g., Bakker 1992; Kats 1939; cf. Nübling 2015).

It is important to note that this ‘age-constraint’ reflects a general tendency rather than a clear-cut rule that all speakers must follow. That is, it is not the case that all speakers use *ziej* for older women and *het* for younger ones; rather, in a previous elicitation study, around one in three speakers did not use the neuter subject pronoun at all and the others varied in the degree to which they used it from under 10 % to over 85 % of the time (Doreleijers et al. 2021; Piepers et al. 2021). These findings were mirrored in follow-up research where again one in three speakers never used *het* at all; this research further showed not only that speakers’ self-reports of using sociopragmatic pronouns varied from ‘never’ to ‘always’, but also that these self-reports generally corresponded well to speakers’ actual language use. Moreover, it was also found that speakers’ attitudes could be related to their pronoun use, and that many – though, importantly, not all – speakers showed relatively high metalinguistic awareness. This suggests that while the sociopragmatic use of pronouns may be conditioned by cognitive automaticity for some speakers, others may take a conscious approach to the appropriateness and/or desirability of the neuter pronoun (Piepers et al. 2023b).

The specific setting in which Limburgian is spoken facilitates variation between speakers, which makes it possible to study the relationships between awareness, automaticity, and use of this pronoun. Limburgian is an officially recognized regional minority language within the Netherlands, although speakers usually refer to it as ‘dialect’ or *plat* ‘vernacular’ (lit. ‘flat’; Bakkes 2002; Louden 2020). This is likely because of the fact that despite its recognition as a regional language, Limburgian is very heterogeneous, comprising many differences across localities, and lacking a standardized form (Bakker 1997). This heterogeneity is facilitated further by the limited role Limburgian plays in formal domains: it is not formally taught in education; in the more formal contexts of everyday life Dutch is often used; and its use in writing tends to be limited to informal domains (e.g., texting or social media; Van Halteren et al. 2018). Dutch is moreover present in the lives of all speakers of Limburgian from a young age, in the form of family members, friends and people encountered in the public domain who do not speak Limburgian, as well as in the media (cf. Cornips 2013: 380; Van den Nieuwenhof et al. 2004). As a result, there is close and continuous contact between Limburgian and Dutch, and all speakers of Limburgian also speak Dutch (Cornips 2013).

### 1.2 Agency in language variation: awareness and attitudes

Social change is often a matter of overcoming automaticity: things we used to do without thinking, unaware of their possibly negative effects, become things we need or want to think about in order to overcome those negative effects. For this to happen, awareness of the effect needs to be raised, and with sufficient awareness it will be possible to stop the automatic production of the problematic behavior, which can then be replaced with desired behavior. This holds true, e.g., for overcoming prejudice (Devine et al. 2012), but as we will argue in this paper, it works in the same way with language, too. Importantly, in the current paper we do not look at concrete instances of language use, and therefore we will not contribute data that directly allow for measuring degrees of cognitive automaticity (or conscious attention); instead, we use data on ‘reported automaticity’: we have asked participants to reflect on how aware they are of their use of a particular feature, a gendered personal pronoun, in their language (see below). For that reason, we will usually refer to ‘reported’ automaticity, to avoid raising the suggestion that we have direct cognitive or neural measurements of automaticity.

‘Agency’ refers, in its most basic meaning, to “the socioculturally mediated capacity to act” (Ahearn 2001: 112) or, similarly, to “the capacity for socially meaningful action” (Parish and Hall 2020: 1). The focus of this paper is on speakers’ agency in language variation. More specifically, our focus is on agency regarding the use or non-use of a specific linguistic item. For the purpose of our current research, we define agency as ‘being able to change one’s language use, based on awareness, and according to one’s attitudes, despite the pressure of automatic production’. It follows that agency, in our view, requires at least two things: (i) awareness of the linguistic item in question; and (ii) an attitude towards it. The first is a prerequisite, as there can be no agency without awareness (Kootstra and Muysken 2019; Squires 2016); the second provides motivation, as going to the trouble of consciously overriding the ease of automatic production is an effort that presumably is only undertaken if there is a good reason to do so (e.g., Thomason 2007).

The notions ‘awareness’ and ‘attitude’ are related: that is, to hold a marked attitude, a speaker needs a level of awareness. Attitudes may generally be overt or covert, the distinction between which is treated in terms of ‘conscious’ and ‘subconscious’, though “the terminology based on “conscious” (…) is preferred to “aware” only for its greater morpho-syntactic flexibility” (Kristiansen 2010: 61). The notions of agency and awareness bring us to Labov’s (1972) distinction between indicators, markers and stereotypes, as well as to Silverstein’s (2003) further development through indexical orders. While first-order indexicality (cf. indicator) indicates linguistic forms that are connected to specific sociolinguistic contexts by linguistic observation from outside a community, second-order indexicality (cf. marker) points to linking that takes place from the inside, i.e., by members of the community themselves. In addition, third-order indexicality (cf. stereotype) indicates the perception of such forms as ‘emblematic’ for a given sociolinguistic context, which are therefore used in stylization practices (cf. Coupland 2001). Shibboleths are recognized not only outside the community (first-order indexicality) but also within the community (second order indexicality) as typical of the community’s language use. By using a shibboleth, speakers show where they are from and as such, shibboleths are part of identification processes (e.g., Busch and Spitzmüller 2021).

Awareness of grammatical choices is not unheard of when the socio-cultural setting calls for it (Aikhenvald 2002; Hill and Hill 1980). When linguistic features become associated with a way of speaking, they become enregistered (Agha 2003; Johnstone 2016). In sociolinguistic work, registers have been investigated as styles of speaking typically tied to particular speakers and particular communicative settings (Agha 2006; Coupland 2001). In language contact settings, register differences have been shown to be sensitive to the degree of purism, i.e., the degree to which one accepts that languages change in conditions of bilingualism (Eisenlohr 2004; Garrett 2005). More in general, contact linguistic studies often show that a purist stance aims to produce language use as devoid of foreign influence as possible (Karrebæk and Ghandchi 2015); a celebratory stance, by contrast, revels in the incorporation of contact effects (Makihara 2005). Many ways of dealing with this dynamic exist in multilingual communities around the world, but what they all have in common is that they involve some degree of awareness of differences between the languages and of what is appropriate language use, fueled by attitudes towards the languages involved and towards language change (see also Kootstra and Muysken 2019).

Importantly, this awareness of linguistic features can affect speakers’ language use, especially in combination with certain attitudes towards these features. For example, research on Catalan showed that young speakers from urban areas did not control the use of *pronoms febles*, ‘weak pronouns’, as well as rural speakers did. According to Woolard et al. (2014]: 131), these “intricate pronominal clitics” are “the frequent focus of sociolinguistic stereotyping, prescriptive commentary, and linguistic-complaint-writing, and of mediatized parodies and humor as well”. Instead of making more mistakes than rural speakers in the use of these pronouns, however, the urban speakers avoided using them altogether by opting for circumlocutions which were likely to have a one-to-one correspondence in Castilian, which is indicative of a high level of metalinguistic awareness (Woolard et al. 2014). Conversely yet similarly, speakers of Sui dialects can be regarded as “performing and constructing identity through the use of linguistic features that index their home communities” despite long-term dialect contact (Stanford 2009: 288). Another example comes from Babel (2011), who shows that in the variety of Bolivian Spanish she studied, Quechua-origin grammatical features are used often in informal speech but not in formal styles. That is, speakers are aware of these features belonging to informal Spanish; whether they are aware of this and actively select them when speaking informally and actively repress them when speaking formally we cannot know – it might be the case that their production in informal style is automatic, just like the production of constructions appropriate of formal Spanish proceeds on autopilot. Finally, Barking et al. (2024) draw attention to the fact that some German speakers who live in the Netherlands actively try to avoid transfer from Dutch: they identify structures which they – correctly or not – attribute to Dutch influence as indicative of a way of speaking German they do not want to be associated with. These examples all suggest that the valence of speakers’ attitudes play a role in language variation, in the sense that a speaker can both avoid and preserve certain linguistic features deliberately (cf. Thomason 2007).

### 1.3 The current study: variation in Limburgian sociopragmatic pronoun use

This paper investigates speakers’ agency specifically in the context of pronoun choice for female referents in Limburgian, a West-Germanic regional language spoken in the Netherlands (see Section 1.1). Crucially, the language has two different personal pronouns which a speaker could use, and speakers vary considerably from one another in whether and how often they use one of these forms. As frequently used elements, which moreover are parts of syntactic paradigms, pronouns are usually produced without much thought (Christiansen and Chater 2016), and conscious attention to them is needed to avoid automatic selection processes – i.e., to achieve agency (Kootstra and Muysken 2019). This paper discusses empirical data showing how Limburgian speakers’ varying metalinguistic awareness of and attitudes towards one of these pronouns may interfere with the cognitive automaticity regarding their self-reported use of this pronoun. Our goal is to map the relationships between metalinguistic awareness, automaticity, and self-reported language use, in order to explain the variation in language use. A language contact setting such as the one in which Limburgian is spoken provides fertile ground for the study of agency, automaticity, and the interplay between them. There are two reasons for this.

First, language contact always takes place in a setting of cultural change, and one of its major consequences is destabilization of the linguistic system, as languages need to respond to the pressures of contact (Aikhenvald 2002; Thomason 2001). In contact settings between a dialect and its cognate standard language, as is the case with Limburgian and Dutch, characteristic dialect features are often replaced by features from the standard language (Auer et al. 2005; Hinskens 2014; Swanenberg and Van Hout 2013). While the use of the neuter form is relatively widespread in Limburgian, it is not a feature that is shared with Dutch: Dutch only uses the feminine pronoun *zij* (cognate with Limburgian *ziej*), while the pronoun *het* (identical in form to Limburgian *het*) is not used for human reference, but instead denotes inanimacy of the referent. In addition, Limburgian *het* is used less frequently in some parts of the region (for more details, see Piepers et al. 2023b), and speakers come in contact with speakers from these areas as well. These circumstances make the feature susceptible to change. Language change is normally only recognized as such if it has spread to a sufficient number of speakers; in the meantime, a change being in progress rather than completed is highlighted by linguistic variation (Backus 2015; Croft 2000; Weinreich et al. 1968). In practice, this means that in language contact settings, there will be a lot of variation in speakers’ language use (Aikhenvald 2002; Thomason 2001).

This brings us to the second point: this variation between speakers can have different underlying reasons (Thomason 2007). If speakers of Limburgian become more used to refer to women with feminine pronouns, for example because that is how it is done in Dutch, they might unconsciously copy that same pattern into their dialect. Convergence towards the societally dominant language Dutch could therefore take the form of *het* being used less often in the dialect, or not at all, as a result of cognitive entrenchment of the ‘Dutch-like’ pattern (automaticity). However, non-use may also follow from speakers attributing more negative meaning to it (pejoration) and therefore avoiding its use (agency). On the other hand, the contrast between Dutch and Limburgian may also spark deviance from the standard language: speakers may also increase the use of *het*, and/or attribute an explicitly positive meaning to it (amelioration). This could, for example, result from a stance that emphasizes the importance of the local dialect and views *het* as an integral and traditional part of Limburgian grammar. Earlier research suggests that in some cases, there is some ‘overuse’ of *het* in contexts where the majority of other speakers would not find it appropriate (Doreleijers et al. 2021), a form of overgeneralization (Doreleijers and Swanenberg 2023; Swanenberg 2020). In this light, *het* may also gain an extra, positive layer of meaning: in addition to any positive meaning of the word itself (i.e., personal closeness, youthfulness), the act of using the word could be associated with authenticity and loyalty to Limburgian language and culture: in other words, the pronoun would be enregistered (Babel 2011; Hill and Hill 1986; Johnstone 2016; Piepers et al. 2023a, 2023b; Stanford 2009).

To summarize, in terms of variation in the use of a specific linguistic item – in our case study, the neuter subject pronoun in reference to women – there are essentially two end results: use, or non-use. However, both the use and non-use of *het* may or may not be a result of a speaker’s agency, and previous research suggests that the main difference would lie in the degree to which speakers have meta- and sociolinguistic awareness regarding the pronoun (Piepers et al. 2023b; cf. e.g., Kristiansen 2010; Nycz 2016; Thomason 2007). Investigating the variation in speakers’ attitudes, metalinguistic awareness, and reported automaticity regarding *het* using self-reports can help understand the variation between speakers’ use of the pronoun. At the same time, studying the relationships between these concepts will help to shed light on the interplay between agency and automaticity in language variation on a more general level. The next section describes how we tackled these issues in the current study.

## 2 Methods

### 2.1 Participants

405 speakers of Limburgian participated in our study: 217 female, 183 male, 3 non-binary, and 2 preferred not to disclose their gender. They ranged in age from 18 to 82 years (*M* = 43.98, SD = 14.99). Participants were recruited through personal communication and social media. Participation was fully voluntary; we raffled off 25 gift cards worth €10 among the participants.

### 2.2 Procedure and outline of the questionnaire

Our research consisted of a questionnaire, in which speakers self-reported on automaticity, attitudes, and metalinguistic awareness (see 2.3). This questionnaire was administered online through *Qualtrics* (2022]). The introduction and instructions, as well as all the questions, were presented in Dutch.1Recall that Limburgian is primarily an oral language, and while it is sometimes written as well, especially relatively formal texts – such as the instructions for and content of our questionnaire – are virtually never encountered in Limburgian (cf. Schmeets and Cornips 2022). We first provided participants with the following background information:2Note that us providing them with this information means there will be no participants who are oblivious regarding *het*. However, in order to be able to ask them focused questions about specific aspects of this pronoun and its use, we had to tell them what the study was about; moreover, we hope to have reduced social desirability bias in participants’ responses by informing them from the outset that we know that variation exists, and letting them know that the statements they would react to had all been uttered by participants in previous studies. We further stressed that there were no wrong answers possible, and urged participants to fill out the questionnaire with the answers that best reflected their personal vision.

“This research project focuses on the special feature of Limburgian that women and girls can be referred to with *het* [neuter] as well as *ziej* [feminine]. Previous research has shown that there are large differences in the intuitions of speakers regarding this. Therefore, the goal of this questionnaire is to map out, on a large scale, how speakers of Limburgian think and feel about the use of *het* for women. To do this, we are going to show you 16 statements. All of these were uttered by participants of our previous research. We would like you to tell us to what extent you agree with these statements.”

Next, we presented the participants with 16 statements (see 2.3 below). After that, we asked them to indicate how often they use *het* in their own language use on a scale from 0 (‘never’) to 10 (‘always’), as well as their age, gender, and the place they grew up in. Finally, participants had the option to add anything that they felt should be noted by the researchers, or anything that they thought should have been included in the questionnaire, but was not.

The respondents all provided informed consent. The questionnaire received ethical clearance from the Research Ethics and Data Management Committee of Tilburg School of Humanities and Digital Sciences at Tilburg University, under ID code REDC2020.144.

### 2.3 Materials

We asked about people’s perception of the three dimensions of pronoun use discussed in Section 1, by presenting them with 16 items in total: (i) automaticity (3 items); (ii) metalinguistic awareness (9 items); and (iii) attitude (4 items). All items contained statements which were uttered by participants of previous research, in an open questionnaire about their intuitions and evaluations of the pronoun (Piepers et al. 2023b). Our current participants reacted to these statements by moving a slider ranging from *Helemaal mee oneens* ‘totally disagree’ (0) to *Helemaal mee eens* ‘totally agree’ (10). Participants were not told about the three themes in the questionnaire; items were presented to them in semi-random order. All participants were shown the same items. We discuss the three dimensions in turn.

#### 2.3.1 Metalinguistic awareness

The first aspect we aimed to measure was *metalinguistic awareness* of the pronoun. This aspect itself consisted of two parts. We asked about their attention to the pronoun and its use in the language as well as daily conversation ((1a–e); the latter two were scored in reverse), to get an idea of the degree to which a speaker notices the pronoun in language use or is even actively thinking about it when speaking.

(1)

a.

**Table:** 

‘When I’m speaking, I have to think about whether I am going to use *het* or *ziej*.’

b.

**Table:** 

‘I notice it when others use *het*.’

c.

**Table:** 

‘I think it is notable that Limburgian and Dutch are so different regarding the use of *het*.’

d.

**Table:** 

‘In a conversation, I am not paying attention to whether my interlocutor uses *het*.’ (reverse)

e.

**Table:** 

‘Before participating in this research, I had never thought about the fact that *het* is used very differently in Limburgian than it is in Dutch.’ (reverse)

Second, we also asked about their awareness regarding general tendencies or norms regarding the use of *het* and how this differs from the feminine pronoun ((2a–d); the latter two were scored in reverse).

(2)

a.

**Table:** 

‘Only when you address someone with their first name, you can call her *het*.’

b.

**Table:** 

‘Older ladies, like for example your grandmother, you should not call *het*.’

c.

**Table:** 

‘To me, *het* means the same as *ziej*.’ (reverse)

d.

**Table:** 

‘The use of *het* for a woman is appropriate in all situations.’ (reverse)

#### 2.3.2 Attitude

The second aspect we aimed to measure was *attitude*, referring to the way in which participants view the pronoun as well as the fact that it exists in Limburgian (examples (3a–d); the latter two were scored in reverse):

(3)

a.

**Table:** 

‘I think *het* is an affectionate form.’

b.

**Table:** 

‘I like the fact that with *het*, Limburgian has a form that Dutch does not have.’

c.

**Table:** 

‘I think *het* sounds degrading.’ (reverse)

d.

**Table:** 

‘I think it is crude to refer to a woman with *het*.’ (reverse)

#### 2.3.3 Self-reported automaticity

The final aspect we aimed to measure was *self-reported automaticity*, which refers to the extent to which respondents feel that their use of the pronoun *het* happens ‘on autopilot’. For this, we used the following three statements, the latter of which was scored in reverse:

(4)

a.

**Table:** 

‘The use of *het* is automatic.’

b.

**Table:** 

‘The use of *het* is embedded in my dialect.’

c.

**Table:** 

‘Using *het* is something that does not come naturally to me.’ (reverse)

### 2.4 Data preparation and analysis

All questions were answered on a scale ranging from 0 to 10 (with two decimal places). We calculated participants’ average score for each set of questions, yielding three final scores (one for each category). The scores for the reversed questions were calculated by subtracting the indicated score from 10. Each participant’s score for *self-reported pronoun use* was also included.

To assess the scales’ reliability, we computed McDonald’s ω (Hayes and Coutts 2020), which was ω = 0.87 for *automaticity* (three items); ω = 0.75 for *attitude* (four items); and ω = 0.52 for *awareness* (nine items). This latter score is on the low side; however, as mentioned, the categories were based on previous research, and the items in the categories were taken from previous participants; therefore, we still included the scale as-is, and will return to this issue of internal consistency in our discussion (see Section 5.3).

To analyze our data set, we performed a moderated mediation analysis (Muller et al. 2005) in *R* using the *JSmediation* package (Batailler et al. 2023; R Core Team 2023), with the following variables (centered): *metalinguistic awareness* as predictor, *self-reported pronoun use* as outcome, *self-reported automaticity* as a mediator, and *attitudes* as a moderator. This analysis is motivated, reported on, and interpreted in Section 3.

Finally, recall that participants had the option to leave an additional comment after having finished the main questionnaire, in case they wanted to elaborate on their answers or explain their thoughts. Many of them did so, and it turned out that these messages often contained a reflection or elaboration on their answers in the questionnaire. We include some of these qualitative additions in Section 4, together with their writers’ scores on the three aspects from the questionnaire, to complement the findings presented in Section 3, and to help better understand the variation between speakers.

## 3 Inferring agency from self-reported metalinguistic awareness, automaticity, and attitudes

We defined agency in this paper as ‘the ability to change one’s language use, based on awareness and according to attitudes, despite automaticity’. That is, we do not directly question the concept of agency itself, but rather aim to infer speakers’ agency from the inter-relationships between three self-reported dimensions of pronoun use and self-reported pronoun use. To do this, we use a moderated mediation analysis, which is a statistical method used to determine whether the strength or direction of a mediating effect (here: automaticity; cf. ‘despite automaticity’) between an independent variable (here: metalinguistic awareness; cf. ‘based on awareness’) and a dependent variable (here: self-reported pronoun use; cf. ‘language use’) is influenced by a third variable (here: attitudes; cf. ‘according to attitudes’). “Moderated mediation happens if the mediating process that is responsible for producing the effect of the treatment on the outcome depends on the value of a moderator variable” (Muller et al. 2005: 854).

**Figure 1:**
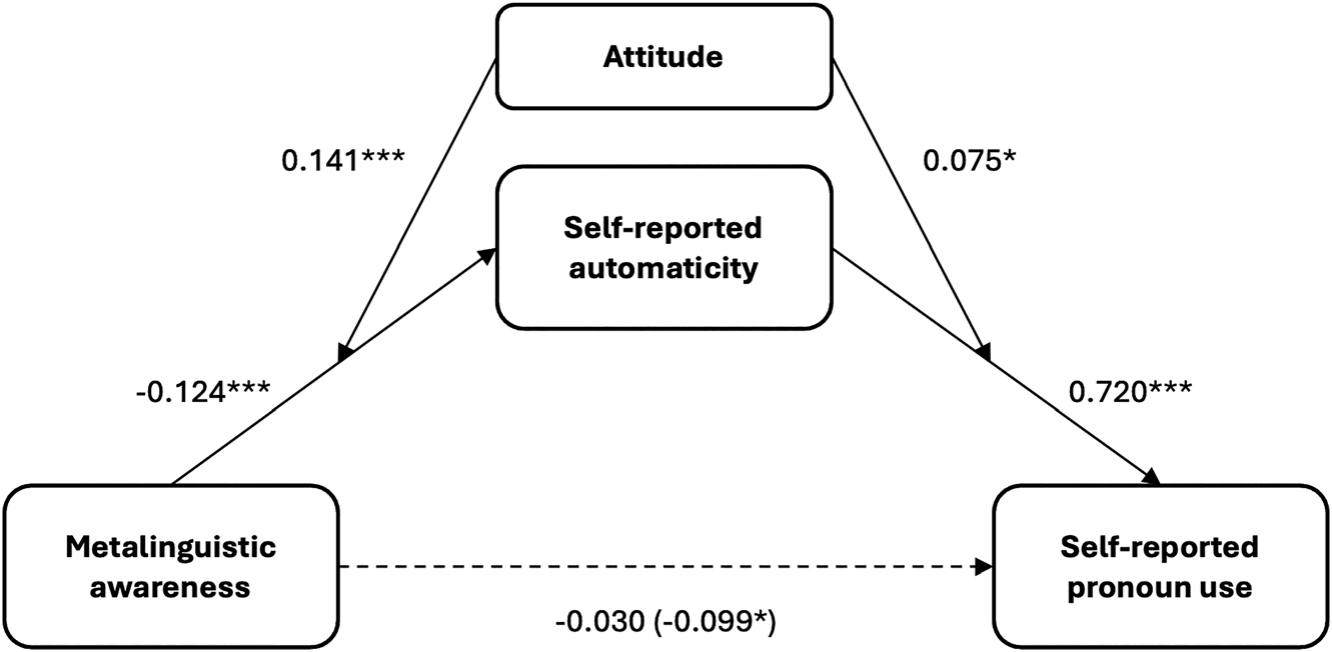
Visualization of the moderated mediation model, with metalinguistic awareness as predictor, reported language use as outcome, automaticity as mediator, and attitude as moderator. The numbers indicate the point estimates for the paths.

To assess whether attitude moderates the indirect effect of metalinguistic awareness on self-reported pronoun use through automaticity, we conducted a moderated mediation analysis in *R* using the *JSmediation* package (Batailler et al. 2023; Muller et al. 2005; R Core Team 2023). Metalinguistic awareness itself did not significantly predict self-reported pronoun use when controlling for the moderating effect of attitude and the mediating effect of reported automaticity, *t*(399) = 0.88, *p* = 0.381. Importantly, however, the analysis revealed that attitude moderated the effect of metalinguistic awareness on reported automaticity, *t*(401) = 4.04, *p* < 0.001, and that attitude also moderated the effect of reported automaticity on reported language use, *t*(399) = 2.25, *p* < 0.05. This pattern suggested the existence of a moderated mediation, which was confirmed by the total stage moderated mediation index, 0.092, 95 % CI [0.041; 0.147] (Monte Carlo simulation, 5,000 2simulations; Yzerbyt et al. 2018). The conceptual model is visualized in Figure 1.

**Figure 2:**
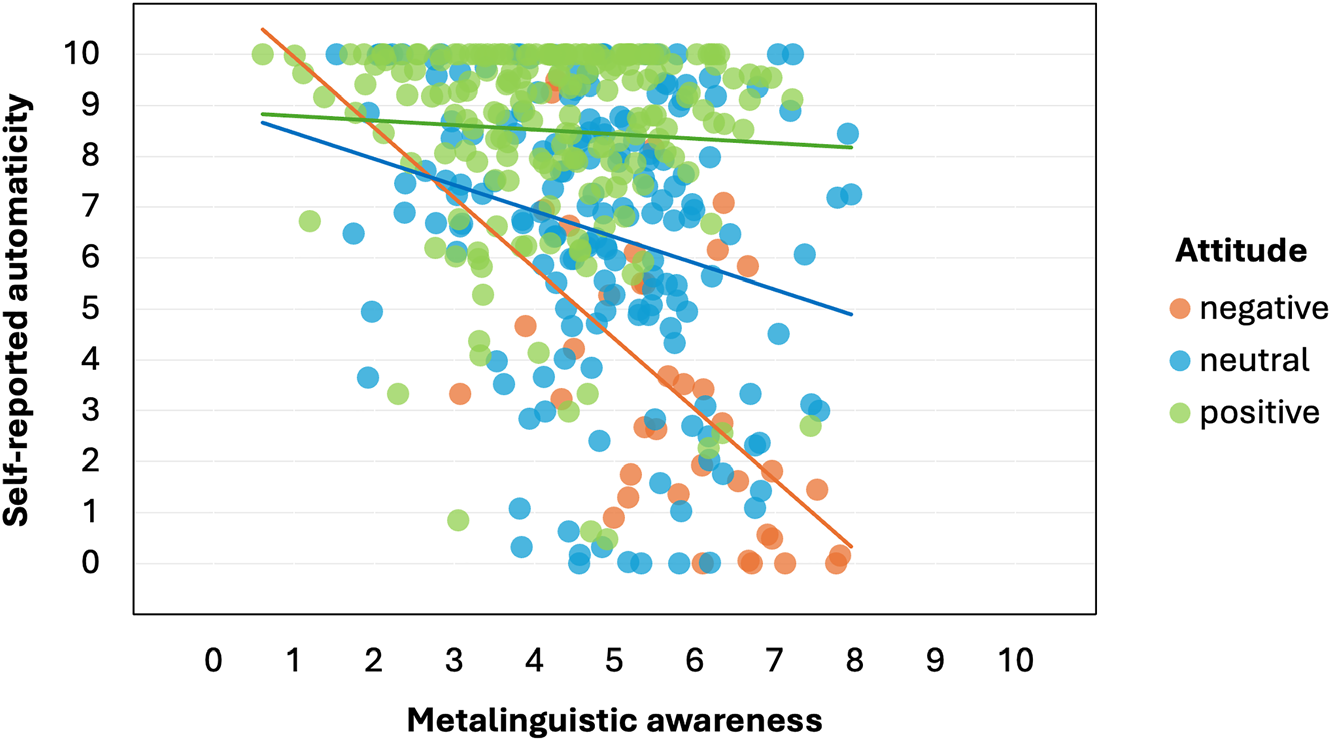
Plot of the relationship between *metalinguistic awareness* and *self-reported automaticity*, varying by *attitude* (categorized).

We interpret this model as follows. First, the mediation of the relationship between awareness and reported language use by reported automaticity shows that automaticity is actually what explains this relationship: that is, if one has a lower metalinguistic awareness of the non-feminine pronoun, then its production will essentially occur largely automatically. Importantly, however, the relationship between metalinguistic awareness and reported automaticity was moderated by attitude, suggesting that while a higher awareness may be able to overrule automaticity (i.e., as it were, ‘turn off’ this autopilot) to some extent, not everyone may want to do this. That is, with a positive attitude towards the pronoun there is no need to intervene in automatic processes regarding the pronoun, while with a negative attitude, this desire may be present, and metalinguistic awareness can lower automaticity. This relationship is illustrated in Figure 2 below. For the purpose of effectively visualizing this relationship for intuitive interpretation, we divided the continuous variable *attitude* into three categories: *negative* (0–3.33), *neutral* (3.34–6.66), and *positive* (6.67–10). Figure 2 clearly shows the interaction between awareness and attitude: higher awareness correlates with lower reported automaticity more strongly for negative attitudes than for neutral attitudes, and more strongly for neutral attitudes than for positive attitudes.

**Figure 3:**
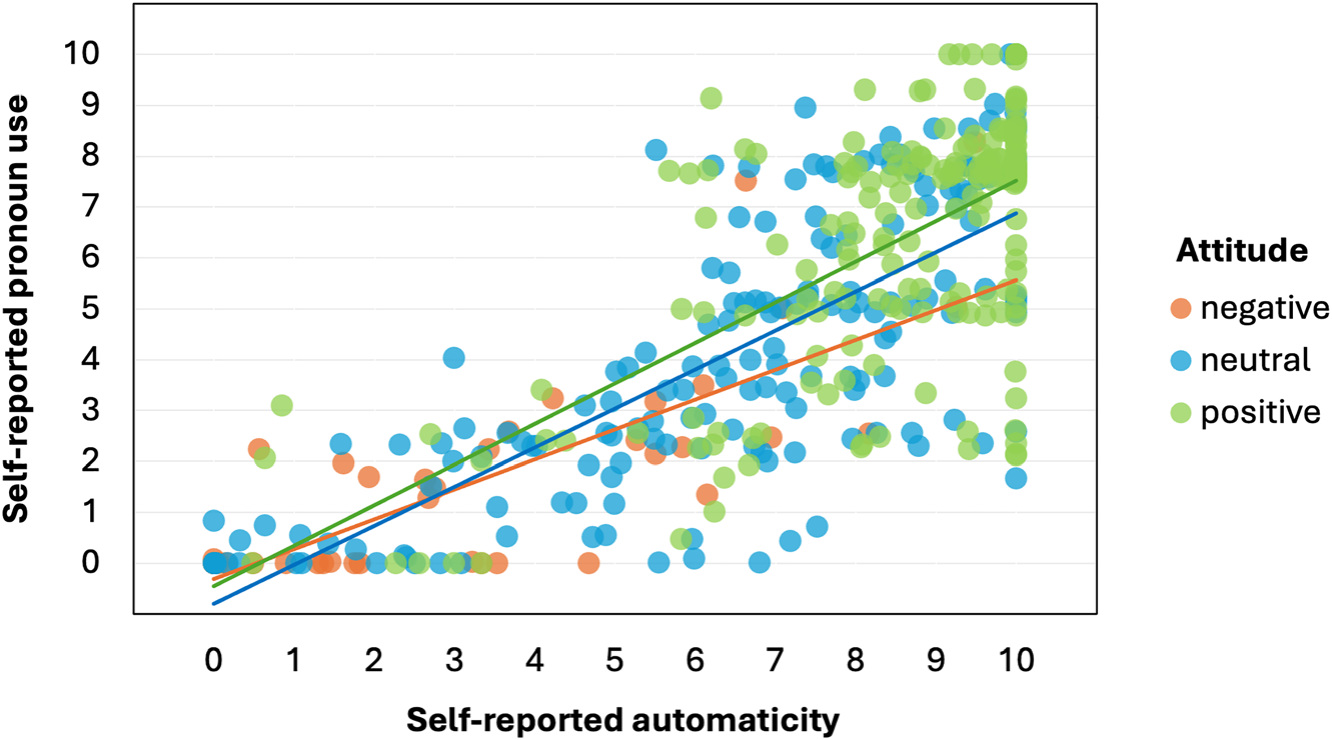
Plot of the relationship between *self-reported automaticity* and *self-reported pronoun use*, varying by *attitude* (categorized).

Reported automaticity, in turn, strongly influenced reported language use, and this effect was further enhanced (i.e. ‘turned up’) by non-conflicting attitudes. This is illustrated in Figure 3 below: the correlation between reported automaticity and reported pronoun use was more positive for positive and neutral attitudes than for negative attitudes.

To summarize, metalinguistic awareness influenced self-reported pronoun use via automaticity, moderated by attitude. Our model showed a negative relationship between metalinguistic awareness and self-reported automaticity, which was moderated by attitude: i.e., if one’s metalinguistic awareness is high, automaticity is likely lower, but automaticity may be boosted (i.e., ‘kept up’) by a positive attitude; conversely, if one’s awareness is low, automaticity is likely to be higher, though this automaticity may be lowered (i.e., ‘overruled’) by a negative attitude towards the pronoun. Since we defined agency as ‘the ability to change one’s language use, based on awareness and according to their attitudes, despite automatic processes’, we interpret the moderating role of attitude as indicative of at least optional agency on the speaker’s part when it comes to the use or non-use of the pronoun *het*. In the next section, we discuss what our respondents had to say about the pronoun use and its various aspects.

## 4 Understanding speakers’ metalinguistic awareness, attitudes, and automaticity

How do people become aware of a linguistic feature? Awareness generally results from “aggregated experiences of in-the-moment noticing of linguistic differences, and coming to understand them as linguistically and socially meaningful” (Squires 2016: 80). From previous research, we know that many – though not all – speakers of Limburgian are able to reflect on various aspects of non-feminine pronoun use (Piepers et al. 2023b). Since we gave participants the option to add anything they wanted to tell us at the end of the questionnaire, we have additional, qualitative data from some participants (i.e., those who chose to answer this optional question). In this section, we present some of their answers in relation to their scores on the questionnaire (note that the answers were given on a scale of 0–10), in order to complement the model presented in the previous section.

First, it should be noted that most of our participants scored high on reported automaticity and attitude but low on awareness, suggesting that many speakers are not all that aware of the pronoun use but do voice positive attitudes when asked about it, and in their pronoun use thus fully rely on automaticity. This is illustrated in (5–6):

(5)

**Table:** 

‘We have been using the word *het* since childhood. It’s ground-in.’
(P157; automaticity 9.29, attitude 7.75, awareness 3.15, self-reported use 10)

(6)

**Table:** 

‘Your everyday language is so natural, you seldom think about it.’
(P173; automaticity 9.88, attitude 7.55, awareness 2.14, self-reported use 7.55)

These speakers discuss how normal and natural the use of *het* is to them. Note, moreover, that while they both describe their own experience, they do not use first person pronouns for this: (5) uses ‘we’, and (6) uses impersonal ‘you’, both suggesting that the experiences described are not exclusive to the speaker, but also assumed to be common to others as well (e.g., Gast et al. 2015).

Second, there was also a fairly large group of participants who scored relatively high on both reported automaticity and awareness, as well as reporting at least moderate (i.e., non-conflicting) attitudes. These speakers, like the previous group, also report to use the pronoun largely automatically, but the main difference is that this group is able to eloquently reflect on the pronoun and its use (cf. Piepers et al. 2023b), as can be seen in (7–8). Similarly, high automaticity and awareness combined with low attitudes also results in metalinguistic reflection (9).

(7)

**Table:** 

‘I have twins, and for me, they are automatically *hae* ‘he’ and *het* ‘she’. I think there is a group of women where both *het* and *ziej* are possible, but that there are also clear groups where really only one of the two is fitting. My grandmother or a female doctor are never *het*, and a child never *ziej*.’
(P36; automaticity 9.25, attitude 8.11, awareness 5.93, self-reported use 6.97)

(8)

**Table:** 

‘For my grandmother, I would use *ziej*, but when I’m talking about an older colleague (around age 60), I would use *het*. Apparently, for me, there is a line somewhere, but I don’t know where that line is yet.’
(P154; automaticity 10, attitude 6.63, awareness 5.53, self-reported use 7.96)

(9)

**Table:** 

‘*Het* comes across as degrading very easily, and can only be used ‘decently’ in certain situations.’
(P224; automaticity 8.16, attitude 2.44, awareness 5.48, self-reported use 2.54)

When a given speaker is highly aware of *het*, the reason for this sometimes is that the speaker has experienced some sort of social situation where their use of or view on *het* did not align with that of others around them (Piepers et al. 2023b). This is referred to as ‘constructed salience’ or ‘top-down salience’, where “an external source provides a context in which something becomes salient” (Boswijk and Coler 2020: 718). This is illustrated in the following examples:

(10)

**Table:** 

‘Now that I live in Brabant, I notice how often I use *het*. My Brabantish husband is only too happy to point this out to me when I’m speaking Limburgian.’
(P186; automaticity 9.41, attitude 5.80, awareness 5.66, self-reported use 8.55)

(11)

**Table:** 

‘I do not live in Limburg, and I have a partner who speaks standard Dutch. Because of this, I am very aware of *het*.’
(P395; automaticity 4.52, attitude 3.82, awareness 7.04, self-reported use 1.18)

In example (10), the context is a bilingual family in which one partner speaks Limburgian and the other does not. Most likely they speak Dutch with each other, in which *het* for women does not occur; yet, in (10), the husband, although not a speaker himself, is apparently attuned enough to his wife’s family’s native dialect to register what is going on pronoun-wise. In this specific case, the speaker did not elaborate on how her husband evaluates this feature – just that he very much likes pointing this out to her, suggesting at least some playfulness – but we know from other speakers in similar situations that their partners sometimes explicitly label it as “very strange” (cf. also Bakkes 2002; Hamans 1989).

A third group of participants reports high awareness, but low reported automaticity and attitudes: that is, they are aware of the feature, but do not like it very much, and do not report to use it. Upon inspection of their qualitative data, these often turned out to be speakers who describe regional differences within Limburgian, discussing how they are from a region where *het* is not used (as much), as in (12–13).3Note that while there are indeed some identifiable regional differences in the frequency of use of non-feminine pronouns across the area in which Limburgian is spoken, regional differences as described by speakers do not necessarily match actual regional patterns (cf. example 38 in Piepers et al. 2023b). There exists a lot of variation even within the same local areas; for an in-depth analysis, see Piepers [to appear]. Similarly, high awareness, low automaticity but a high attitude might also be found in this group (14), as might low scores on automatically and awareness as well as attitudes (15):

(12)

**Table:** 

‘My wife is from Susteren, and she does use *het*.’
(P197; automaticity 0, attitude 0, awareness 7.77, self-reported use 0)

(13)

**Table:** 

‘There is a big difference between northern Limburg, and the south and middle parts. In the northern part the word *het* is not used for women; it’s used from Venlo southwards.’
(P242; automaticity 1.30, attitude 3.03, awareness 5.16, self-reported use 0)

(14)

**Table:** 

‘In the Meijel dialect, there is no *het*. I was raised with two dialects, and therefore also speak the dialect of Helden. In that case, I do sometimes use *het*.’
(P47; automaticity 2.69, attitude 7.59, awareness 7.44, self-reported use 2.53)

(15)

**Table:** 

‘In Maastricht the word *het* is seldom used.’
(P217; automaticity 3.33, attitude 2.84, awareness 3.07, self-reported use 0)

Fourth, low reported automaticity and awareness combined with high attitudes turned out to be found in speakers who do not use the word *het* specifically, but report on using another non-feminine subject form instead (for a more elaborate analysis of this specific phenomenon, see Piepers et al. 2023a), as in (16) (note again the use of ‘we’). Similarly, there was also a participant who reported using both non-feminine subject forms (i.e., *het* and *hae*), and consequently differs from the other in automaticity regarding *het* (17):

(16)

**Table:** 

‘We use a different word: *hae* ‘he’. *Hae* is for both women and men! We rarely ever use *het*.’
(P82; automaticity 4.37, attitude 8.74, awareness 3.31, self-reported use 2.40)

(17)

**Table:** 

‘It’s also normal for me to say *hae* when I refer to a woman, even though its literal translation is ‘he’. Still, it’s normal to do that in my dialect.’
(P75; automaticity 10; attitude 7.04, awareness 4.40, self-reported use 7.77)

We now briefly return to the contrast between Limburgian and Dutch. We already showed in some previous examples how speakers may be aware of this contrast, as well as the interpretational ‘clash’ this may invoke in a contact setting. Similar awareness of this contrast is shown in (18).

(18)

**Table:** 

‘In my answers, I based myself on situations in which I speak Limburgian, but with, for example, the statements “I think it is crude to refer to a woman with ‘het’” and “I think ‘het’ sounds degrading” I would answer ‘totally agree’ for Dutch.’
(P35; automaticity 9.49, attitude 7.57, awareness 5.22, self-reported use 9.32)

While the participant in (19) is also aware of this contrast, this person expresses a very different stance, conveying the wish that the use of *het* would become acceptable in Dutch as well:

(19)

**Table:** 

‘I identify as non-binary and don’t like being called *ziej*, but *het* feels better to me. It’s a shame that Dutch doesn’t have this option!’
(P295; automaticity 9.16, attitude 9.83, awareness 5.91, self-reported use 7.61)

Other speakers, however, may not be aware of this contrast, the contact situation between Dutch and Limburgian, or even the structural similarities between the languages. Example (20) presents a qualitative addition from a speaker who suggests that comparing the two languages is silly. This speaker does not seem to consider that there is, in fact, close and continuous contact between Limburgian and Dutch, and makes an apples-and-oranges comparison with other, seemingly randomly chosen languages, to make their point. This speaker reports high automaticity and use, a moderate attitude, and low awareness.

(20)

**Table:** 

‘I think it is strange that Limburgian is compared to Dutch. Why? I would say, ‘two languages, two entities’. You wouldn’t say that it is strange that French or Farsi have different pronouns than Yiddish, either?’
(P130; automaticity 9.99, attitude 5.01, awareness 2.17, self-reported use 10)

Finally, and unsurprisingly, we find speakers who explicitly mention their views of this pronoun changing or having changed after participating in our research. It appears that the speaker in (21) may have experienced a slight shift in attitude regarding *het*:

(21)

**Table:** 

‘I have thought about the different use of *het* in Limburgian before, but it was only after this questionnaire that I realized that it is indeed quite anti-social, or could be perceived that way.’
(P296; automaticity 9.99, attitude 6.25, awareness 2.04, self-reported use 8.85)

All in all, the sample quotes presented in this section provide insight into how speakers view and think about the pronoun *het* and which aspect of this (i.e., automaticity, attitude, or awareness) stands out to them, as indicated by which part they choose to comment on.

## 5 Discussion

The purpose of this study was to contribute to finding out to what extent linguistic ‘choices’ are made automatically or through conscious selection of linguistic units. While it is clear that both autopilot and conscious choice play a role whenever we talk, it is difficult or impossible, outside controlled laboratory settings, to know for sure which mechanism a speaker used when producing specific parts of an utterance, from the smallest morpheme to larger multi-word units. In this study, speakers were not put into a lab setting, nor did they produce spontaneous speech; instead, we asked them about the degree to which they think they produce a specific feature (a subject pronoun) in general in a specific language (the non-written Germanic regional language Limburgian, spoken in the Southeastern part of the Netherlands). The study is part of a larger research project in which this pronominal use is investigated more widely; here, we focus on the degree to which we can say something about the interplay between automatic production of this form, general metalinguistic awareness people show about the various forms they could choose between and when they are generally used, by others and by themselves, and the attitudes people have towards these forms.

### 5.1 Agency and automaticity in pronoun use

In Section 3, we reported evidence for a statistically significant moderated mediation model, with *metalinguistic awareness* as predictor, *self-reported pronoun use* as outcome, *self-reported automaticity* as mediator, and *attitude* as moderator. This model shows that the use of the pronoun *het* is most strongly driven by automaticity. However, metalinguistic awareness combined with a negative attitude can lower automatic production of the pronoun, even right down to zero usage; similarly, automatic use may be further boosted by positive attitudes. That is, while *het* is mostly used automatically, speakers can overrule this automaticity if they want to. In our introduction, we defined ‘agency’ as ‘being able to change one’s language use, based on awareness and according to their attitudes, despite automatic processes’; our results suggest that speakers can indeed do this, at least to some extent.

Importantly, however, various combinations of scores for the individual categories occur, and although we do find a statistically significant model, this does not mean that this model captures all types of variation between speakers. Moreover, the question of whether a speaker at a specific moment uses a specific linguistic item consciously or unconsciously remains, unsurprisingly, unanswered – we simply do not know. With morphemes which always need to be used and show no variation (e.g. the first person pronoun for first person reference) automaticity is likely to be by far the most frequently used mechanism of production. For the inherently variable reference to third person female referents in Limburgian, it has proven insightful to study how automaticity and agency relate to one another in order to understand variation between speakers. Looking at the qualitative data in Section 4, although not collected systematically in this research (but see Piepers et al. 2023b), helps understanding the various differences between speakers in how and why they react a certain way to the pronoun, and underlines the fact that no two language users are the same (cf. Dąbrowska 2012, 2020]).

### 5.2 Awareness and attitudes in a language contact setting

The general idea that cognitive automaticity is the driving force behind language production is clearly reflected in our data. However, if metalinguistic awareness – together with attitudes – does have the power to reduce the cognitive automaticity with which personal pronouns are generally assumed to be produced, attitudes could be argued to be a mechanism which could, in principle, initiate a change in usage. This is especially true for sociopragmatic pronouns, since the factors – both grammatical and socio-pragmatic – which guide their use appear to be robust and fickle at the same time; that is, the pronouns appear well-entrenched on the one hand, yet it is also clear that speakers hold different opinions, and that these opinions can be very strong. To some extent, this variability is inherent to sociopragmatic gender, as the underlying categorization is relatively vague, and the use and interpretation of sociopragmatic pronouns is largely dependent on speakers’ assessments of interpersonal relationships (see Piepers et al. 2023b).

In a language contact situation in which a standard language exerts influence on a closely related regional language or dialect, linguistic changes can sometimes be harder to identify than in contact situations where the languages involved are typologically further apart and features can be straightforwardly attributed as belonging to one language or the other. This is analogous to what is observable when one mixes colors: it is easier to observe that mixing yellow and blue results in the color green, than it is to spot that mixing red and orange yields a slightly darker shade of orange. Limburgian and Dutch are closely related and structurally similar: the pronoun *het* exists in both languages, in the same form and with the same syntactic function. However, only in Limburgian does it have the sociopragmatically governed use for referencing female referents, and as a result, a possible effect of Dutch on Limburgian *het* should be observable in speakers who do not use it in this function, and in the presence of overt attitudes towards the pronoun. Similarly, but harder to prove, would be changes in the rate with which the pronoun is used and with which referents.

Especially when combined with negative attitudes towards *het*, non-use of the neuter pronoun could be seen as a clear example of a deliberate change, an ‘act of identity’ (cf. Kristiansen 2010), and it is likely that this accurately describes the linguistic behavior of some of our participants. On the other hand, linguistic changes often come about naturally and inadvertently, as the result of subconscious attitudes (Kristiansen 2010). Given the complex pattern of reasons people have for their use or non-use of *het* that arises from the moderated mediation analysis, it seems likely that both conscious and subconscious attitudes play a role, with an as yet relatively low presence of overt attitudes (that would strongly drive the deliberate change, or ‘change from above’, referenced above). The substantial impact of automaticity, though, illustrates that people in general are creatures of habit. Items (i.e., words, multi-word units, or constructions) which are used and encountered often are well-entrenched for them; in turn, well-entrenched items are the ones they use often, because these are more easily activated (Backus 2014; Bybee 2010). It could therefore be expected that speakers who use Dutch a lot in their daily lives might consequently use Limburgian *het* less. We did not systematically investigate the impact of Dutch on Limburgian, and are therefore not in a position to draw conclusions regarding the extent to which the former influences the latter. However, what we can conclude is that it is not simply the case that all speakers who hear and/or use more Dutch consequently make less use of *het* in their Limburgian (see also Piepers et al. 2024). Examples (10) and (11) came from two speakers in a similar situation: both no longer live in Limburg, and both have a partner who does not speak Limburgian, which has heightened their awareness. The first speaker’s use of *het* does not seem to have lowered; her reported automaticity is still high, and her attitude is above average. The second speaker, however, reports a considerably lower use, automaticity, and attitude.

Among speakers of Limburgian, there appears to be a general acceptance of linguistic variation: speakers know that others might not speak in the same way they do, and they generally do not mind (see Piepers et al. 2023b). However, this acceptance does seem largely limited to *regional* differences; that is, regional variation is socially acceptable and even celebrated, but generational differences, as a result of a changing social landscape where the standard language plays an increasingly larger role (cf. Backus and Swanenberg 2013), are often viewed in a less favorable light (see (22)).

(22)

**Table:** 

‘I have lived in the *Randstad* [an urban area in the western part of the Netherlands] since 1970, and I speak a conserved dialect. An old lady once interrupted me in the middle of a conversation, telling me “what a beautiful dialect you speak, that’s how people used to talk, you don’t hear that anymore nowadays”. I still have regular discussions with my brother and sister about what ‘correct dialect’ is. I think I notice changes in my brother’s dialect that I do not notice in my sister’s.’
(P405; automaticity 10, attitude 6.37, awareness 7.23, self-reported use 5.13)

An individual speaker’s journey from a state of no or low metalinguistic awareness to high(er) awareness has consequences for language use, and, since people differ from one another regarding their attitudes and the degree to which these attitudes play a role in language use, these consequences are unpredictable. However, this unpredictability does not mean there are no identifiable patterns: that is, it is not the patterns themselves which are unpredictable; rather, what is hard is anticipating which pattern a given speaker is going to follow. This echoes the discussion put forward by e.g., Swanenberg (2020) that speakers may react differently to a changing dialect, and dialect change thus might produce contrasting results, leading to more variation across speakers.

The question that needs addressing is why we find so much variation. The current study supports earlier findings (Piepers et al. 2021, 2023a, 2023b, 2024]) that uncovered similar variation in how Limburgian speakers convey third person reference to women. One could have expected different patterns, for example widespread use of the Dutch pattern (only feminine pronouns), or the generational pattern familiar from Heritage Languages (the younger the speaker, the more use of the Dutch pattern). Instead, we found a pattern of variation that seems to be governed by the degree to which speakers *want* to change this aspect of Limburgian grammar. Speakers with no or neutral attitudes continue to speak the way automaticity guides them to do, preserving entrenched lexico-grammatical usage. For them, the use of socio-pragmatically conditioned non-feminine pronouns in Limburgian is entrenched enough to not be dislodged by their presumably equally entrenched use of the Dutch equivalent of their feminine third person pronoun (recall that most Limburgian speakers communicate in monolingual Dutch settings on an everyday basis). They hold transfer at bay, presumably mostly as an automatic process, but in some cases also because they have positive attitudes towards aspects of Limburgian that make the language different from its otherwise very similar powerful neighbor.

If one were to write a grammar of Modern Limburgian, this would produce a dilemma: how to present the Limburgian pronominal system? Apart from the regional variation (some regions make less frequent use of the non-feminine pronoun), there is the social variation we have documented in this study. Grammars are, of course, always abstractions that up to a point have to gloss over differences between individual speakers, who may or may not make use of the constructions presented as part of the language’s grammar. However, the variation in pronoun use is substantial, and makes it difficult to say at the moment what the grammatical ‘rules’ are. Limburgian is, in this sense, typical of minority languages everywhere: contact produces changes, and changes trigger attitudes. While many contact-induced changes are more or less uniformly adopted given enough time and intense enough contact, this uniformity is halted when attitudes play a role. Essentially, this means some speakers manage to overcome automaticity in such cases. Some speakers aim for this, others do not. In many minority languages, this dynamic plays out as an opposition between a modernist stance and a purist stance (e.g., Aikhenvald 2003; Hill and Hill 1986; Makihara 2005; see also Aalberse et al. 2019). Our qualitative data suggest it is more complex than this, since having a negative attitude to the non-feminine pronouns does not necessarily mean that a speaker avoids them. That is, although many people clearly have opinions about the pronoun, it is not the case that the feature has become widely seen as indexing a particular persona. The seeds for such enregisterment (cf. Agha 2003; Babel 2011) are there, and for some speakers the process is well under way; for many others it seems not to have started; yet others are aware of this incipient enregisterment but resist it. The concept of ‘enregisterment’ is clearly useful here, especially since it describes a process rather than an end result, and the pronominal system we are investigating seems to be in flux.

Returning once more to the hypothetical grammar writer faced with the task of describing the pronominal system of Limburgian, and therefore predicting what an individual speaker will say when faced with the task of referring to a woman in the third person, this grammarian would have to accept that the usage is actually unpredictable. Two different ways of referring are acceptable in general (continuing to use sociopragmatic gender, including the natural variation due to personal patterns in who to refer to with *het* and who with *ziej*, or establishing the uniform use of the feminine pronoun), but this acceptability is not uniform. The differences in attitudes we found in our sample show this. A further dimension of variation is that for some speakers, these attitudes come with an intention to act: they show agency and avoid the pronoun they see as maintaining an inherent sexism in the language (or, in some cases, use it proudly as a badge of Limburgian identity). For them, choice of pronoun is a socially meaningful act, which means that for them metalinguistic awareness of pronoun choice is high enough that they can avoid the gravitational pull of automaticity that seems to govern the bulk of the speakers in our sample.

### 5.3 Methodological considerations

One major point of discussion regarding our approach is the fact that we asked speakers about our concepts of study instead of directly measuring our variables. That is, we collected no language production data, but instead asked participants to report their own pronoun production, and we also derived the concepts of automaticity, awareness and attitude from answers instead of directly measuring them cognitively. The clear advantage of this approach is that it allowed us to collect data from many participants; the downside, however, is that we have to fully rely on the self-reports of these participants in our interpretation. This comes with some important caveats, which we discuss in turn.

First, the cognitive automaticity of a linguistic unit is generally expressed in degrees of *entrenchment*, which is often inferred from language production based on e.g., frequency counts of the unit itself and of its co-occurring linguistic and sociolinguistic elements (i.e. units are often part of larger constructional units and may be typically used in particular sociolinguistic settings), and aspects of communicative settings that may increase or decrease the unit’s salience (e.g., Backus 2005; Bybee 2010; Divjak 2019). The idea is that a linguistic item becomes entrenched for a speaker through both active use and passive exposure: an item which is used and encountered more often is more entrenched, and this higher degree of entrenchment in turn leads to easier activation and thus more frequent use of the item in language production when communicative and social triggers call for it. Our approach was to ask speakers about their perception of the extent to which they used the pronoun. Asking speakers for self-reports regarding automaticity obviously yields different data than direct measurements of the pronoun’s frequency in their speech; what we hope to have shown, however, is that such self-report data do allow for interesting insights into an important aspect of automaticity: the degree to which speakers feel they can consciously attend to an aspect of language use that might, on the one hand, be seen as highly entrenched and thus easily activated without conscious attention, and, on the other hand, as socio-pragmatically salient and thus triggering conscious attention. Clearly, the variation this produces would not be visible from frequency counts in language use. If a speaker feels that the pronoun might not fit every context and therefore uses it less often, in the cases that it *is* used, it can still come about naturally for this speaker (i.e. entrenched and therefore easily activated). This appears to be the case for e.g., the speaker in (22), who reports high automaticity, yet moderate use. For future research, it would be desirable to find ways to combine data on reported automaticity with direct measures of an item’s cognitive automaticity as well as of its frequency in language production of the same speaker, to avoid having to put all eggs in the basket of language users’ self-reports.

Second, metalinguistic awareness has proven to be a tricky, multifaceted concept. One basic issue with its inclusion in our questionnaire is the fact that including it in the first place inevitably has *caused* awareness, yielding some intrusive version of the observer effect, with unknown and unpredictable effects on further language use of our respondents (see example (21)). A more substantive issue, however, concerns the way in which we included awareness in our questionnaire (cf. the low ω). Awareness regarding Limburgian *het* can exist on two separate yet related levels: first, speakers may or may not be aware of the pronoun’s use for women within the language and the contrast this creates with the contact language Dutch; second, they may or may not be aware of ‘rules’ or patterns regarding the pronoun’s appropriateness in Limburgian. In addition, the questions are open to at least two interpretations. For example, a low score for e.g., the statement “Only when you address someone with their first name, you can call her ‘het’” could essentially mean one of two things: *(i)* the respondent does not agree, i.e., for them, *het* may also be used for people with whom they are not on a first-name basis; or *(ii)* they did not know that it may be viewed as an inappropriate pronoun for people with whom they are not on a first-name basis. That is, we cannot be sure whether individual respondents’ low scores reflect unawareness of the pattern, or a conscious breach of it.

Third, attitudes, too, are likely more multifaceted than we are able to capture in the current questionnaire. Because speakers are so different in how they view the pronoun *het* (see Piepers et al. 2023b for an elaborate discussion of this), it is very much possible that they will also differ from one another in the weight they assign to certain aspects of it, and how they interpret it, leading to similar issues as described above. That is, if an individual respondent views *het* as an affectionate form and does not at all view it as degrading, this respondent will get a very high score for attitude. If another respondent then does not view the pronoun as affectionate, but also not as degrading, this second respondent will get a lower score on attitude than the first one; however, this does not necessarily mean that the first respondent actually views *het* more positive in everyday language use – it may just mean that the second respondent simply does not see *het* as specifically affectionate, which does not really have to do anything with their attitude in general.

## 6 Conclusions

In this paper, we investigated speakers’ reported awareness, attitudes, and automaticity regarding the pronoun *het* in Limburgian, and how these relate to self-reported use. Our findings suggest that automaticity is the driving force in the use of this pronoun, but this autopilot may be curbed by awareness and attitudes. That is, if one’s awareness is high, reported automaticity is likely lower, but automaticity may be boosted (i.e. ‘kept up’) by a positive attitude; conversely, if one’s awareness is on the lower side, reported automaticity is likely to be higher, though this automaticity may still be lowered (i.e. ‘overruled’) by a negative attitude towards the pronoun *het*. While our focus was on one particular lexical-grammatical item, we feel this is in keeping with a general usage-based view on language use as continuously combining the relatively fast and automatic production of material that does not need much conscious attention and the relatively intentional selection of material needed to convey information in precisely the way that best fits current socio-communicative intentions. Importantly, our results also showed that speakers may not only differ in terms of automaticity, awareness, and attitudes, but also in the importance they assign to either of these aspects. Combining quantitative data with qualitative data helps understanding various differences between speakers in how and why they react a certain way to one and the same pronoun. We conclude that while cognitive automaticity does appear to be the general driver of language use in this domain, it is possible to overrule this process if speakers are both able and interested. Our findings highlight that language users differ from each other, and speakers may be affected differently by language contact situations, with various effects in their individual language use.
